# Macrophage Migration Inhibitory Factor Is Involved in Ectopic Endometrial Tissue Growth and Peritoneal-Endometrial Tissue Interaction *In Vivo*: A Plausible Link to Endometriosis Development

**DOI:** 10.1371/journal.pone.0110434

**Published:** 2014-10-17

**Authors:** Halima Rakhila, Karine Girard, Mathieu Leboeuf, Madeleine Lemyre, Ali Akoum

**Affiliations:** 1 Endocrinologie de la Reproduction, Centre Hospitalier Universitaire de Québec, Québec, Québec, Canada; 2 Département d'obstétrique et gynécologie, Faculté de médecine de l'Université Laval, Québec, Québec, Canada; Center for Molecular Biotechnology, Italy

## Abstract

Pelvic inflammation is a hallmark of endometriosis pathogenesis and a major cause of the disease's symptoms. Abnormal immune and inflammatory changes may not only contribute to endometriosis-major symptoms, but also contribute to ectopic endometrial tissue growth and endometriosis development. A major pro-inflammatory factors found elevated in peritoneal fluid of women with endometriosis and to be overexpressed in peritoneal fluid macrophages and active, highly vascularized and early stage endometriotic lesions, macrophage migration inhibitory factor (MIF) appeared to induce angiogenic and inflammatory and estrogen producing phenotypes in endometriotic cells *in vitro* and to be a possible therapeutic target *in vivo*. Using a mouse model where MIF-knock out (KO) mice received intra-peritoneal injection of endometrial tissue from MIF-KO or syngeneic wild type (WT) mice and *vice versa*, our current study revealed that MIF genetic depletion resulted in a marked reduction ectopic endometrial tissue growth, a disrupted tissue structure and a significant down regulation of the expression of major inflammatory (cyclooxygenease-2), cell adhesion (αv and β3 integrins), survival (B-cell lymphoma-2) and angiogenic (vascular endothelial cell growth) factorsrelevant to endometriosis pathogenesis, whereas MIF add-back to MIF-KO mice significantly restored endometriosis-like lesions number and size. Interestingly, cross-experiments revealed that MIF presence in both endometrial and peritoneal host tissues is required for ectopic endometrial tissue growth and pointed to its involvement in endometrial-peritoneal interactions. This study provides compelling evidence for the role of MIF in endometriosis development and its possible interest for a targeted treatment of endometriosis.

## Introduction

Endometriosis is an estrogen-dependent inflammatory disease defined by the presence of endometrial glands and stroma outside the uterine cavity. Although multiple theories exist regarding the etiology of endometriosis, the implantation hypothesis is the most commonly accepted [1]. Shed endometrial fragments are believed to adhere to peritoneal surfaces, proliferate and invade deeply into the subperitoneal space. A variety of genetic, hormonal, immune and inherent endometrial changes may support the development of endometriosis lesions within the peritoneum of affected women and the progression of the disease. Immune-modulating factors produced either by immune or endometrial cells, were shown to be involved in the abnormal capability of endometrial tissue to attach to, invade and grow into host tissues [2]–[4]. The available literature points to an important role for macrophage migration inhibitory factor (MIF). Beyond its eponymous effect on activating or inhibiting macrophage mobility, MIF is now understood as a major pro-inflammatory factor and a critical upstream regulator of innate immunity [5], [6]. Studies with MIF-deficient mice confirmed an upstream activating role for MIF in diverse inflammatory responses in the host response to infection and tumorigenesis [7], [8]. Furthermore, in addition to directly inducing angiogenesis, MIF helps, thereby increasing the release of other growth and angiogenic factors [9]–[13].

Our previous studies showed a positive correlation between MIF levels and endometriosis. MIF expression is particularly elevated in active and early stage endometriotic lesions and significantly upregulated in eutopic endometrium from women with endometriosis, where it varies with the stage of the disease and correlates with its major clinical symptoms, namely infertility and pain [14], [15]. MIF levels are elevated in the peritoneal fluid and peripheral blood of women with endometriosis as well and MIF secretion is enhanced in the peritoneal macrophages of these patients [13], [16], [17]. The available literature supports our findings [18]–[20], which further revealed the capability of this key pro-inflammatory cytokine to promote angiogenesis direct and indirect pathways, activate cell proliferation, stimulate the synthesis of prostaglandin E_2_ (PGE_2_), and trigger local estradiol synthesis in endometriosis stromal cells [9], [11], [14], [21]–[23].

However, other than an associational relationship, it is unclear if this cytokine effectively plays an active role in the development and/or progression of endometriosis. Based on the current literature and our previous *in vitro* and *in vivo* observations, we addressed the role of MIF in establishing endometriosis-like lesions using knockout (KO) genetic approaches. Here we demonstrate that MIF absence markedly impaired ectopic endometrial tissue growth and implantation in the peritoneal cavity of MIF-KO animals, while a specific inhibition of MIF in Wild-Type (WT) mice significantly reduce the extent of the disease. Furthermore, add-back of MIF to MIF-KO animals led to a significant increase in the number and size of endometriosis-like lesions.

## Materials and Methods

### Animals

All procedures involving animal experimentation were approved by the “Comité de protection des animaux du CHUQ”(permit ID: 2012139-01). *In vivo* experiments were performed according to the Canadian committee of animal's protection rules. Female WT BALB/c andC.129S4 (B6)-Miftm1Dvd/Jmice were purchased from Jackson Laboratory (Bar Harbor, Maine, USA). MIF-KO mice are homozygous for a mutation in the MIF gene mapped to chromosome 10 [7], [24]. To remain inbred, our strain was maintained by sibling mating or, if necessary, by parent-offspring mating. Genotyping was performed for each experimental mouse according to the manufacturer's protocol. All mice were housed under pathogen free conditions and a 12-hour dark/light cycle. Mice were given food and water *ad libitum*.

### *In vivo* model of endometriosis

WT mice: The murine model used in this study has been described in details elsewhere [25]. WT mice (6–8 weeks old) challenged with endometrium (n = 6) underwent ovariectomy at day -6 and were injected intramuscularly (IM) with 100 µg/kg of estradiol (E_2_) valerate in corn oil (Sigma, St. Louis, MO, USA) at days -6, 0, 6, and 12. E_2_ supplementation was carried out to abrogate differences related to the stages of the estrous cycle. Syngeneic WT mice used as donors (6–8 weeks old) also underwent ovariectomy and E_2_ treatment at day-6, but they were sacrificed at day 0. Donor mice (one donor for two recipients) were killed via isoflurane/O_2_ (3–4%) (Abbot Laboratories, Saint-Laurent, Quebec, Canada) inhalation and then euthanized in a CO_2_ chamber (10 l/min). Uteri were removed and placed in sterile phosphate-buffered saline solution (PBS). The serosal and myometrial layers were dissected under stereomicroscopy and the remaining endometrium was placed in PBS, finely minced. Tissue homogenates were pooled, pelleted by centrifugation, resuspended in 1 mL PBS, labeled with carboxyfluorescein diacetate, succinimidylester (CFDA-SE) (10 µM) (Life Technologies Inc., Burlington, ON, Canada) during 20 min at room temperature and examined under a fluorescence stereomicroscope to confirm labeling.

Recipient animals were given an analgesic solution of buprenorphine (0.3 mg/mL) by intradermal injection and anesthetized with a mixture of isoflurane/O_2_ (3–4%). Endometrial homogenates were washed twice and re-suspended in PBS (200 µL/uterine horn). Each recipient mouse received an equal quantity of endometrial homogenates (equivalent to one uterine horn) via intraperitoneal (IP) injection [26], [27]. Twelve days post-inoculation, animals were anesthetized with isoflurane/O_2_ (3–4%), then euthanized in a CO_2_ chamber (10 L/min) and the abdominal cavity of each mouse was explored using a fluorescence stereomicroscope equipped with a green fluorescent protein (GFP) filter for *in situ* identification, localization, enumeration and measurement (in two perpendicular diameters) of endometriosis-like lesions using Micro-Imaging GmbH stereomicroscope (Carl Zeiss, Jena, Deutschland).

MIF-KO mice: For these experiments, both MIF-KO (MIF^−/−^) and syngeneic WT mice (positive controls) served as donors and recipients. In cross experiments, KO mice were used as donors and WT mice as recipients, and *vice versa*. Mice (n = 6/group) received I.M.100 µg/kg of E_2_ valerate in corn oil6 days before induction of endometriosis and every 6 days afterwards. Killing of donors, tissue processing and labeling, and induction of experimental endometriosis in recipient mice was performed as described above.

### Pharmacological approaches

On day 12 following inoculation of endometrial tissue, KO mice inoculated with KO endometrial tissue (n  =  5) received daily and for 6 consecutive days I.P. injection of ISO-1 (4 mg/kg in 100 µL PBS) [26], [28], [29] or 100 µL PBS (control group, n  =  5). ISO-1 or (S,R) 3-(4-hydroxyphenyl)-4-5-dihydro-5-isoxazole acetic methyl ester) is defined as a highly specific inhibitor to the catalytic site of MIF. In parallel, KO mice inoculated with KO endometrial tissue (n  =  5) were treated for 6 consecutive days with I.P. injection of rhMIF (0.008 mg/kg in 100 µL PBS) [30], [31] or with I.P. injection of 100 µL PBS (control group, n  =  5).

### Macroscopic and histological analyses

The number, size and location of endometriosis-like lesions were identified by fluorescence stereomicroscopy and endometriosis-like lesions were photographed *in situ* using an Axiocam camera and Axiovisio Rel 4.8 software (Carl Zeiss, Jena, Deutschland). Endometriosis-like lesions were dissected from surrounding tissue, fixed in 10% formalin (Fisher scientific, New Jersey, USA) and then embedded into paraffin. Serial 4 µm tissue sections were rehydrated and stained with hematoxylin and eosin. For immunostaining, tissues were rinsed in cold PBS and treated with 3% hydrogen peroxide to block endogenous peroxidase activity.

To detect cytokeratin-7, sections were incubated overnight at 4°C with rabbit polyclonal anti-human cytokeratin-7 antibody (GeneTex, Irvine, Canada) [1∶3200 dilution in PBS/bovine serum albumin (BSA) 0.02%/Tween-20 0.05%]. To detect CD74, MIF receptor, and proliferating cell nuclear antigen (PCNA), sections were incubated for 90 min at room temperature with a rabbit polyclonal anti-human CD74 (GeneTex, Irvine, Canada) [1∶1600 dilution in PBS/bovine serum albumin (BSA) 0.02%/Tween-20 0.05%] or a rabbit polyclonal anti-human PCNA (GeneTex, Irvine, Canada) [1∶800 dilution in PBS/bovine serum albumin (BSA) 0.02%/Tween-20 0.05%]. Sections were then incubated for 1 hour at room temperature with peroxidase-conjugated goat anti-rabbit antibody (Jackson ImmunoResearch Laboratories, Mississauga, Canada) [1∶500 dilution in PBS/BSA 0.02%/Tween-20 0.05%] before being incubated with the peroxidase substrate 3, 3′-diaminobenzidine for 5 min at room temperature, rinsed in PBS, counterstained with hematoxylin and mounted in Mowiol.

### Molecular analyses

RNA extraction and quantitative real-time polymerase chain reaction (qRT-PCR): Endometriosis-like lesions were dissected under fluorescence stereomicroscopy from the surrounding tissue using iris scissors and stored at −80°C until assay. Total RNA was extracted using Trizol reagent (Life Technologies Inc.) according to the manufacturer's instructions. Total RNA concentration was measured by densitometry (260/280 nm) using a NanoDrop spectrophotometer, RNA was reverse transcribed and stored at −80°C until PCR. To quantify mRNA levels of mouse glyceraldehyde-3-phosphate dehydrogenase (GAPDH), integrin αV (ITGAV), integrin β3 (ITGB3), cyclooxygenases (COX) 1and 2, B-cell lymphoma-2 (BCL2), BCL2-associated X protein (BAX) and vascular endothelial growth factor (VEGF), qRT-PCR was carried out using an ABI 7500 Thermal Cycler (Applied Biosystems, Foster City, Canada) according to the manufacturer's instructions. Each standard PCR reaction contained 2 µL of reverse transcriptase product, 0.5 µL of each primer (final concentration, 0.033 µg/µL), 10.5 µLRNase and DNase-free water, 12.5 µL SYBR Green PCR Master Mix, which contains Taq DNA polymerase reaction buffer, dNTP mix, MgCl_2_, SYBR Green, and Taq DNA polymerase. To ensure that the detected product resulted from amplification of cDNA rather than from contaminating genomic DNA, primers were selected and designed with Primer Premier 5 software to cross intron-exon boundaries. The primer sequences, the reaction conditions and the sizes of the amplified fragments are listed in Table 1. For every evaluated genes, the mRNA level in each sample was normalized GAPDH mRNA level. After each run, melting curve analysis (55°–95°C) was performed to verify the specificity of the PCR reaction. All samples were run in duplicate and each run included negative controls without RNA and without reverse transcriptase.

**Table 1:** Primer sequences for RT-qPCR.

Gene (mouse)	Sequence	Product size (bp)	Tm (°C)	Gene bank accession number #
BAX		160	58	NM_007527.3
Forward	5′-GGCGAATTGGAGATGAACTG-3′			
Reverse	5′- GCAAAGTAGAAGAGGGCAACC-3′			
BCL2		170	60	NM_009741.3
Forward	5′-GTCACAGAGGGGCTACGAGT-3′			
Reverse	5′-CAGCGGTGGCAACGAG-3′			
COX2		318	59	NM_011198.3
Forward	5′-GCTGTACAAGCAGTGGCAAA-3′			
Reverse	5′-GCTCGGCTTCCAGTATTGAG -3′			
ITGAV		101	60	NM_008402.3
Forward	5′-CGAGGGAAGTTACTTCGGATTC-3′			
Reverse	5′-GCTGGGTCGTGTTCGCTTT-3′			
ITGB3		84	60	NM_016780.2
Forward	5′-TGCGTCCGCTACAAAGGG-3′			
Reverse	5′-GCCAGTCCAGTCCGAGTCAC-3′			
VEGF		123	60	NM_001025250.3
Forward	5′-TAACGATGAAGCCCTGGAGTG-3′			
Reverse	5′-CATCTGCTGTGCTGTAGGAAGC-3′			
GAPDH		131	60	NM_008084.2
Forward	5′-CCTTCCGTGTTCCTACCCC-3′			
Reverse	5′-GCCCAAGATGCCCTTCAGT-3′			

### Statistical analysis

Data related to the evolution of the body weight followed a nonparametric distribution and were analyzed using the Kruskal-Wallis test. Data related to the number and size of endometriosis-like lesions and qRT-PCR followed a Gaussian distribution and were analyzed using the unpaired Student's *t*-test for two comparisons or ANOVA and the Bonferroni's test *post-hoc* for multiple comparisons (GraphPad Software, San Diego, CA). Differences were considered as statistically significant for p <0.05.

## Results

### MIF absence impedes ectopic endometrial tissue development

**Figure 1:**
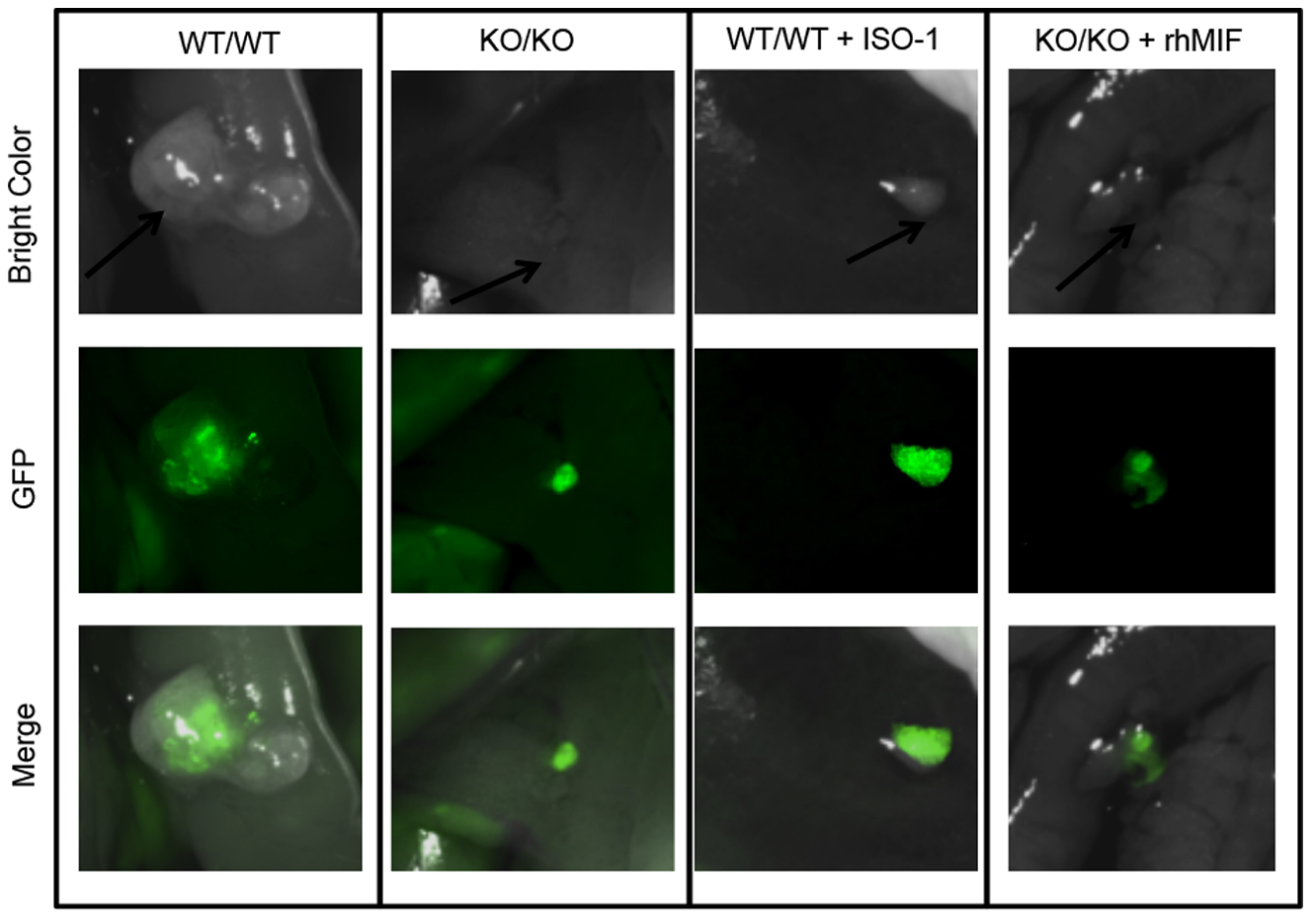
Stereomicroscopic observation of endometriosis-like lesions. Endometrial implants as labeled with CFDA-SE and observed at sacrifice by fluorescence stereomicroscopy in MIF-KO mice inoculated with endometrial tissue from syngeneic MIF-KO mice (KO/KO) or WT mice inoculated with syngeneic WT murine endometrial tissue (WT/WT) (controls). Endometrial implants in KO/KO mice treated with rhMIF (0.008 mg/kg) or WT/WT mice treated with ISO-1 (4 mg/kg) are shown. Implants were observed by optic light or epi-fluorescence (GFP). Merged images are shown.

**Figure 2:**
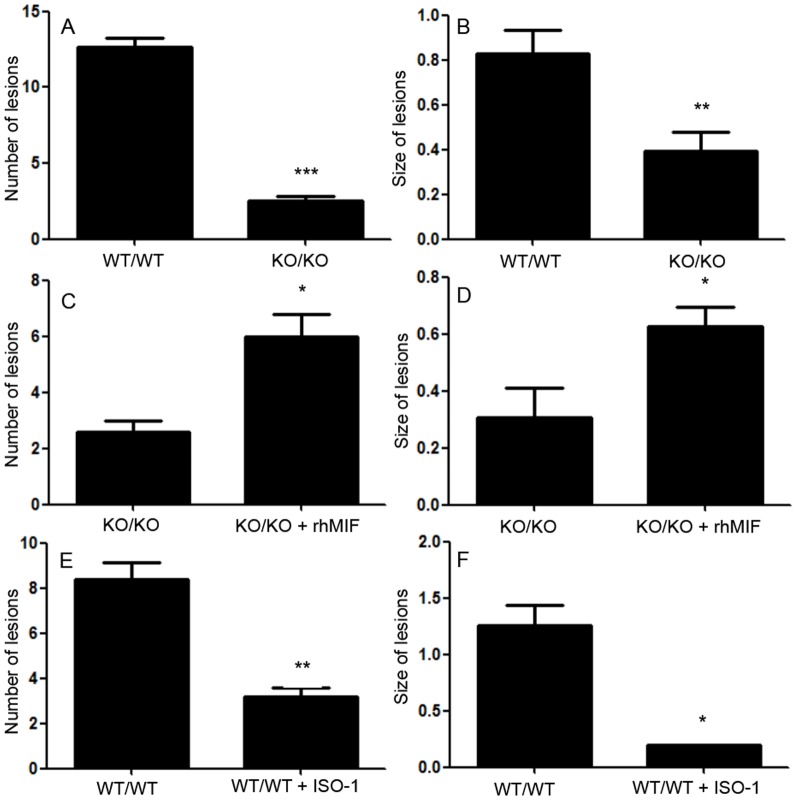
Development of endometriosis-like lesions in KO/KO mice and control WT/WT mice. Endometriosis-like lesions in KO/KO mice and control WT/WT mice were numbered and their sizes measured under fluorescence stereomicroscopy at sacrifice (A, B). Parallel experiments were performed where rhMIF (0.008 mg/kg) was added back to KO/KO mice (C, D), while ISO-1 (4 mg/kg) was used to treat WT/WT mice (E, F). Data are from 5 or 6 mice per group and expressed as mean ± SEM;*, p< 0.05, **, p< 0.01 and ***, p< 0.001 compared with the corresponding control group with the unpaired t-test.

In this model, mouse endometrial tissue was allowed to implant and establish into the peritoneal cavity of recipient mice, and endometriosis-like lesions were localized and examined under fluorescence stereomicroscopy (Figure 1). In experiments where WT mice were donors and recipients (WT/WT) (controls), all mice developed endometriosis-like lesions within 12 days. The mean number and size of lesions were 12.7± 0.5 and 0.8 ± 0.1 mm^2^, respectively. However, when KO mice were used as donors and recipients of endometrial tissue (KO/KO), 80% less endometriosis-like lesions (2.5 ± 0.3, p< 0.001) were found in comparison with control mice (Figure 2A). Furthermore, these lesions were significantly smaller than those found in control mice (0.39 ± 0.2 mm^2^, p< 0.01) (Figures 1 and 2B).

Parallel experiments were also carried out where WT mice received endometrial tissue from KO and *vice versa*. Our data displayed in Table 2 showed that the number of endometriosis-like lesions was significantly decreased compared to the control group (6.0 ± 0.6and 4.8 ± 0.6, respectively) (p< 0.001). However, no differences in the number or size of endometriotic lesions were observed between these two groups.

**Table 2:** Development of endometriosis-like lesions in KO mice *versus* WT mice.

Donor	Recipient	Lesion number		Lesion size	
		Mean ± SEM [Range]	Percent change (%)	Mean (mm^2^) ± SEM	Percent change (%)
WT	WT	12.7 ± 0.5 [11]–[15]	0	0.83 ± 0.1	0
KO	KO	2.5 ± 0.3^c^ [1]–[3]	−80	0.39 ± 0.2^b^	−53
WT	KO	6.0 ± 0.6^c^ [3]–[8]	−52	0.49 ± 0.3	−40
KO	WT	4.8 ± 0.6^c^ [3]–[7]	−62	0.78 ± 0.2	−6

WT, wild type mice; KO, MIF knock out mice.

Data are from 6 mice per group and expressed as mean ± SEM.

b p< 0.01 and ^c^p< 0.001, compared with control (WT mice receiving WT endometrial tissue).

It is noteworthy that endometriosis-like lesions were located on the peritoneum, intestines, spleen, liver and uterus-adnexa, but no significant differences in the number or size of endometriotic lesions between these different peritoneal implantation sites were found (data not shown).

### MIF add-back to KO donor/recipient mice improves the number and size of endometrial implants

The role of MIF was further assessed by treating KO donor/recipient mice with rhMIF. As shown in Figures 1 and 2 and Table 3, MIF add-back improved endometrial tissue growth and resulted in a significant increase in the number of endometriotic lesions(6.0± 0.8; p < 0.05), as well as in the size of these lesions (0.60± 0.06; p < 0.05) as compared to control mice treated with vehicle (2.6 ± 0.4 and 0.30 ± 0.10 mm^2^, respectively).

**Table 3:** Effect of rhMIF add-back in KO mice or specific inhibition of MIF in WT mice on the development of endometriosis-like lesions.

Donor	Recipient	Lesion number		Lesion size	
		Mean ± SEM [Range]	Percent change (%)	Mean (mm^2^) ± SEM	Percent Change (%)
KO	KO + Vehicle	2.6 ± 0.4 [2]–[4]	0	0.30 ± 0.10	0
KO	KO + rhMIF	6.0 ± 0.8^a^ [3]–[7]	+130	0.60 ± 0.06^a^	+93
WT	MIF^+/+^ + Vehicle	8.4 ± 0.7 [6]–[10]	0	1.25 ± 0.17	0
WT	WT + ISO-1	3.2 ± 0.4^b^ [2]–[4]	−61	0.19 ± 0.01^a^	−84

Wild type (WT) mice; MIF knock out (KO) mice; rhMIF, KOmice treated with rhMIF; ISO-1, WTmice treated with ISO-1; Vehicle, mice treated with PBS instead of rhMIF in KOmice or ISO-1 in WTmice. Data are from 5 mice per group and expressed as mean ± SEM.

a p< 0.05 and ^b^p< 0.01 compared with the corresponding vehicle-treated group.

### Treatment with ISO-1 reduces the number and size of endometriosis-like lesions

In order to confirm the involvement of MIF in ectopic endometrial tissue development, we performed experiments where control WT/WT mice were treated with a selective specific inhibitor of MIF (ISO-1). As shown in Figures 1 and 2 and Table 3, treatment with ISO-1 significantly reduced the number (3.2 ± 0.4, p < 0.01) and size (0.19 ± 0.01 mm^2^, p < 0.05) of endometriotic-like lesions as compared to control mice treated with the vehicle alone (8.4 ± 0.7 and 1.25 ± 0.17 mm^2^, respectively).

### Treatment with rhMIF or ISO-1 has no negative impact on the animals' weight and survival

Pharmacological treatments had no perceptible effect on the health of these animals.

**Figure 3:**
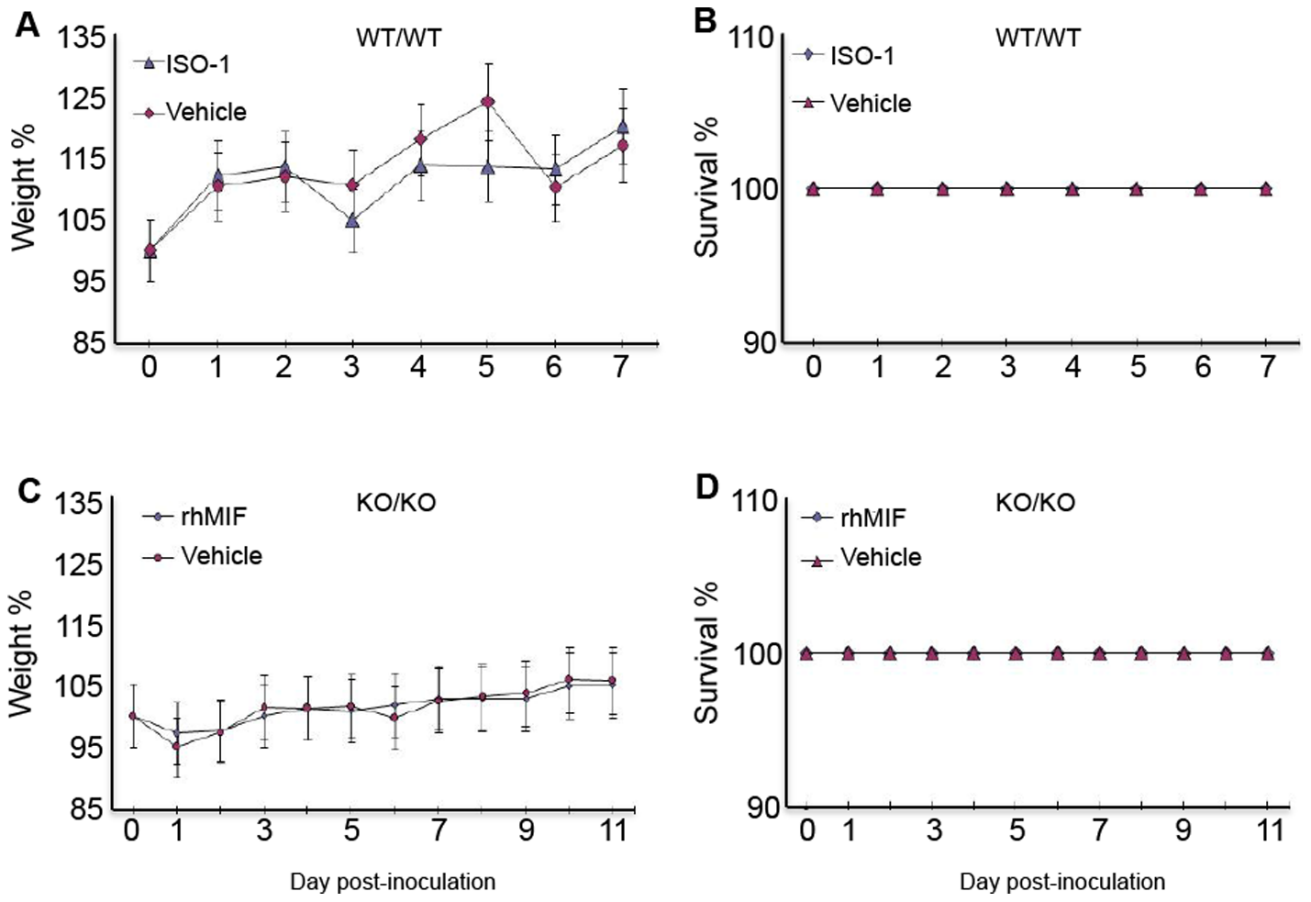
Effect of endometrial tissue inoculation and treatment on the body weight and survival rate of animals. Mice were treated with ISO-1 (4 mg/kg) (A, B), rhMIF (0.008 mg/kg) (C, D). Control mice were treated with the vehicle (PBS). Data are means ± SEM from 5 mice treated with ISO-1, 5 mice treated with rhMIF and 10 mice treated with the vehicle.

On the first hand, animals exposed to rhMIF, ISO-1or vehicles showed no significant weight loss or gain. On the other hand, all mice survived after treatment with 0.008 mg/kg of rhMIF or 4 mg/kg of ISO-1. The results are displayed in Figure 3.

### MIF absence disrupts endometriosis-like tissue structureand causes atrophy and necrosis

**Figure 4:**
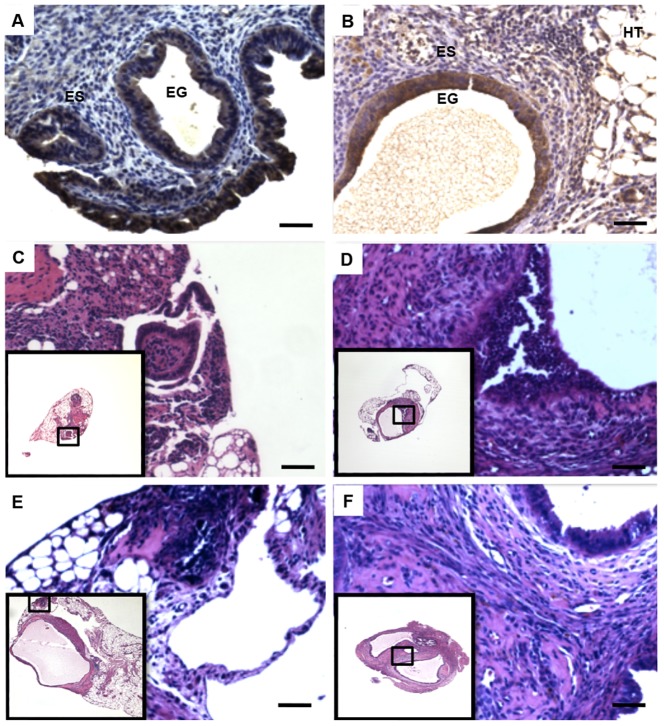
Immunostaining and histological examination of murine endometrial implants. Immunostaining of endometrial tissue from WT mice before (A, black arrows) and after inoculation and implantation into WT mice (controls) (B, black arrows) showing CK7-positive epithelial glands. ES, endometrial stroma; EG, endometrial glands;HT, host tissue. Hematoxylin-eosin staining of endometrial implants from KO/KO mice(C), WT/WT mice (D) and mice treated with ISO-1 (E) or rhMIF (F). Insets show general histological views of endometrial implants. Scale bar,10 µm.

Histological analysis of endometriosis-like lesions in WT/WT control mice prior to any treatment showed a well-preserved endometrial tissue, which was surrounded by peritoneal host tissue and displayed endometrial stromal and glandular components similar to those of the initial murine endometrial tissue. Endometrial glands were identified by immunostaining of an epithelial cell marker (cytokeratin-7) (Figure 4, A and B).

**Figure 5:**
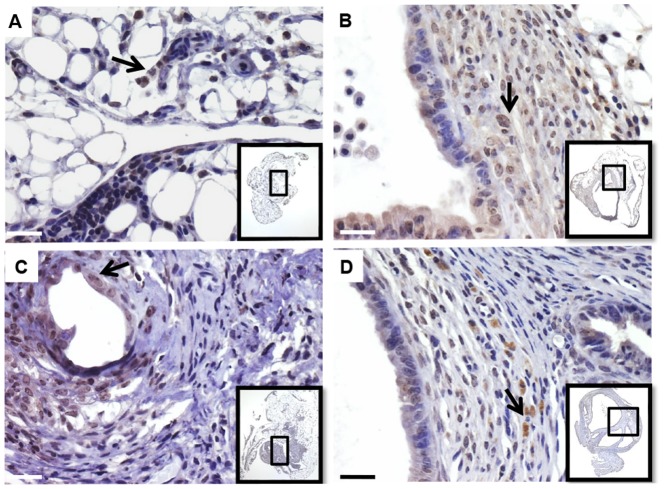
Immunostaining of MIF receptor CD74 in murine endometrial implants. CD74 immunostaining was carried out on implants from KO/KO mice (A), WT/WT mice (B) and mice treated with ISO-1 (C) or rhMIF (D). Insets show general histological views of endometrial implants. Black arrows show CD74 positive cells. Scale bar, 10 µm.

Histological assessment of endometrial implants developed in KO/KO mice showed an atrophied and necrotized tissue that was mostly replaced by dense connective tissue (Figure 4C). This contrasts with endometrial implants found in WT/WT mice, which rather exhibited columnar glandular endometrial cells surrounded by loose connective tissue similar to endometrial stroma, and foci of proliferating epithelial cells (Figure 4D). Histological evaluation further showed disrupted structures in endometrial implants in mice treated with ISO-1, whereas implants from KO/KO mice treated with rhMIF showed well-defined endometrial glands and stroma surrounded by host mouse tissue (Figure 4, E and F). In addition, we showed that endometriosis-like lesions and mouse endometrial tissue before inoculation (data not shown)were receptive to MIF because of the expression of its receptor CD74, as analyzed by immunohistochemistry (Figure 5).

### MIF knock-out or inhibition significantly affects molecular pathways involved in the development of endometriosis-like lesions

**Figure 6:**
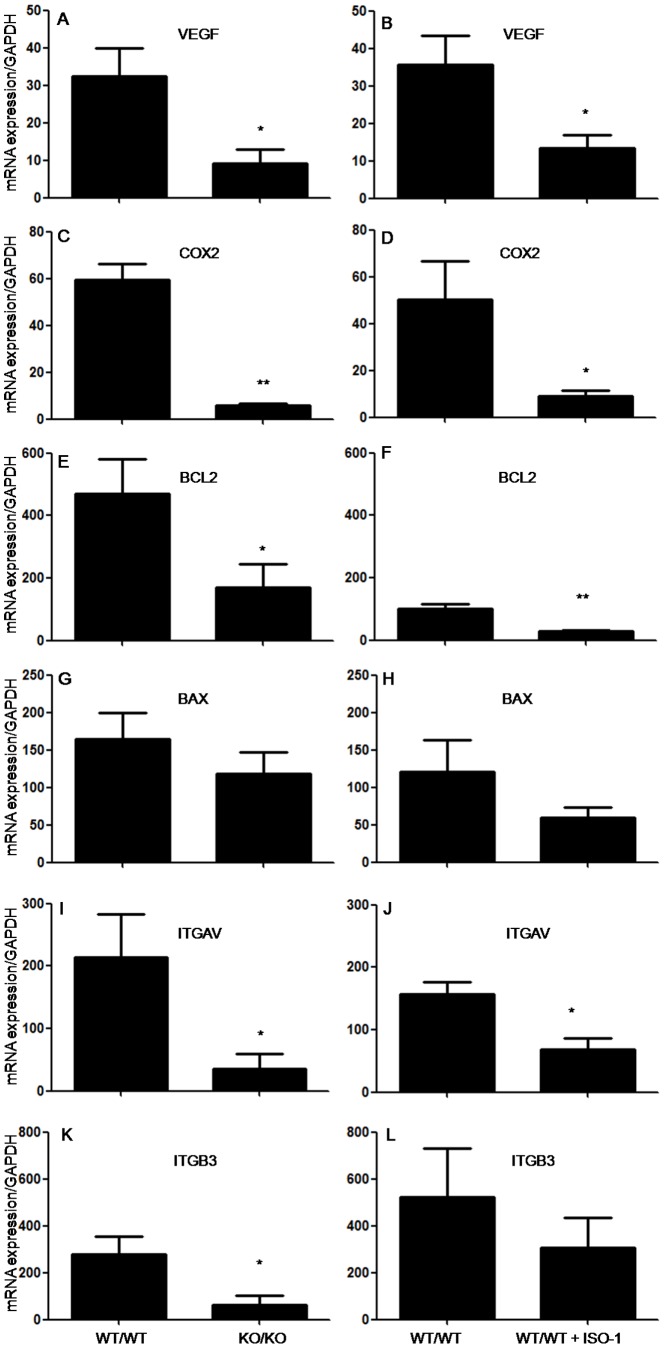
Real-time PCR analysis of the expression of genes mainly involved in cell adhesion and vascularisation of endometriosis-like lesions. Histogram representation of the effect of MIF genetic depletion or antagonism *versus* controls on VEGF (A, B), COX2 (C, D), BCL2 (E, F), BAX (G, H), ITGAV (I, J) and ITGB3 (K, L) mRNA expression in endometriosis-like lesions by quantitative real time PCR. For each factor, the ratio of mRNA level to GAPDH mRNA was determined. Results were from WT and KOmice (n  =  6) with no treatment (controls) and from WT treated with ISO-1 (n  =  5). Data are mean ± SEM; *, p <0.05 and **, p < 0.01 as compared to the control group.

**Figure 7:**
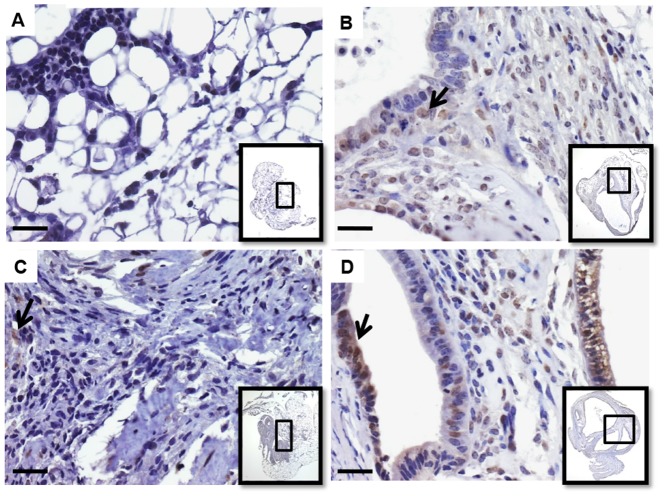
Immunostaining of PCNA in murine endometrial implants. PCNA immunostainingwas carried out on endometrial implants from KO/KO mice (A), WT/WT mice (B) and mice treated with ISO-1 (C) or rhMIF (D). Insets show general histological views of endometrial implants. Black arrows show CD74 positive cells. Scale bar,10 µm.

A complex network of biological processes is involved in the aptitude of endometrial tissue to grow into ectopic sites and develop endometriosis, including cell adhesion, survival and angiogenesis. To understand the molecular pathways that may underlie the marked negative impact of MIF absence or the selective chemical inhibition of MIF on ectopic endometrial tissue growth observed *in vivo*, we further assessed the expression of main inflammatory, adhesion, angiogenesis and cell survival/apoptosis mediators found to be abnormally expressed in human endometriotic lesions. Data exhibited in Figure 6, A and B, shows that VEGF, a major angiogenic factor found to be upregulated in active endometriotic lesions [32], is down-regulated in endometrial implants from KO/KO mice and those from WT/WT mice treated with ISO-1 (p < 0.05 and p < 0.05, respectively). Since COX2 catalyzes the production of PGE_2_, a main inflammatory mediator of inflammation and angiogenesis, and was shown to be highly expressed in human endometriotic lesions [33], [34], we assessed it expression levels and found a significant down-regulation in both KO/KO and ISO-1-treated WT/WT mice models (p < 0.01and p < 0.05, respectively) (Figure 6, C and D). Our data further showed that the expression of BCL2, which is known for promoting cell survival, was significantly decreased, in KO/KO mice (p < 0.05)and WT/WT mice treated with ISO-1 (p < 0.01) (Figure 6, E and F). On the other hand, the expression BAX, a BCL2 protein family member that induces apoptosis by antagonizing BCL2 proapoptotic function, did not show any statistically significant changes in both models (Figure 6, G and H). KO/KO mice showed a significant inhibition in the expression of adhesion receptors ITGAV and ITGB3 which are well-known to regulate cell adhesion, growth, survival and gene expression [35] (p< 0.05) (Figure 6, I and K), but only ITGAV was significantly decreased in ISO-1-treated WT/WT (Figure 6, J and L). In order to confirm these results, we assessed the expression of PCNA, a marker of cell proliferation, in the different combination of endometriosis-like lesions. As shown in Figure 7, PCNA labeling was noticeably low in lesions from KO/KO and ISO-1-treated WT/WT mice, as compared to WT/WT and rhMIF-treated mice.

## Discussion

This study provides evidence that MIF promotes ectopic endometrial tissue growth and development *in vivo*. Using a MIF-KO model and a control WT mouse model of endometriosis, where syngeneic endometrial tissue was injected into the peritoneal cavity and allowed to implant and grow, our data first showed that MIF absence markedly reduced endometrial tissue growth into the peritoneal host tissue and resulted in a significant diminution of the number and size of endometrial implants. Interestingly, selective inhibition of MIF using a specific inhibitor (ISO-1) in WT mice led to comparable outcomes, whereas MIF-add back to MIF-KO animals significantly increased endometriosis-like lesions' size and number. The action of MIF could be explained by the expression of its receptor, CD74, which was expressed in endometriosis-like lesions. Histological and molecular analyses of endometrial implants corroborated the macroscopic findings and showed that genetic MIF depletion or specific chemical blockade of MIF disrupted endometrial tissue structure, led to noticeable tissue atrophy and necrosis and down-regulated the expression of VEGF, COX2, BCL2 and integrins αv and β3, which are key mediators of angiogenesis, inflammation, adhesion and cell survival.

These findings are of relevance to endometriosis pathophysiology considering the biological functions of MIF and our current knowledge of the disease. Essentially, MIF is a potent multifunctional proinflammatory, angiogenic [10], [22], [36]–[38], immune-modulatory and growth-promoting factor [6], [39]. MIF is also quite known for inhibiting apoptosis, favoring cell survival and stimulating proteolysis, tissue remodeling and tumor invasion [40]–[43]. According to our data, MIF stimulates COX2 expression in ectopic endometrial cells and elicits a pro-angiogenic and pro-inflammatory phenotype [11], [21], which may potentiate the capability of these cells to stimulate the host angiogenic response and exacerbate the immune-inflammatory reaction occurring in the implantation site. Although considered as a benign gynecologic disease, endometriosis is believed to mainly result from the abnormal survival, adhesion, invasion and growth of endometrial tissue into extra-uterine sites, primarily in the peritoneal cavity. Peritoneal inflammation is a major hallmark of endometriosis and the available evidence supports a significant role for immune and inflammatory factors in the disease's major symptoms and the ability of endometrial tissue to implant and develop endometriosis [3], [44], [45]. MIF, a major proinflammatory factor, may represent a key component of the local inflammatory network that develops in the peritoneal implantation sites. MIF expression levels are increased in peritoneal macrophages and endometriotic lesions [13], [14] and its concentrations are elevated in the peritoneal fluid [16]. MIF may play an important role in the retention of activated peritoneal macrophages [46], stimulation of endometrial cell adhesion[22], induction of angiogenic phenotype in ectopic endometrial cells [11], up-regulation of MMP activity [43]and promotion of cell survival [40], [47], [48].

Our current data showed that MIF genetic depletion affects ectopic endometrial tissue growth and had a significant impact on several biological pathways closely associated with the pathogenesis of endometriosis. These findings are substantiated by the significant impact of MIF blockade with a specific chemical inhibitor shown in the current syngeneic WT mouse model and consistent with our previous findings with established human endometriosis in nude mice [26]. Thus, the lack of MIF led to a decreased expression of VEGF and COX2 in endometriosis-like lesions. This is quite interesting considering the elevated expression of these factors in endometriotic lesions, particularly in active, early-stages and highly vascularized lesions [14], [32], [33] and our previous *in vitro* data showing the capability of MIF to stimulate the secretion of potent angiogenic and inflammatory factors including VEGF in human endometriotic cells and upregulate COX2, the rate-limiting enzyme for prostaglandin (PG) synthesis, and PGE_2_ release [11], [21]. Beside their well-documented role as potent mediators of inflammation and pain, PGs are endowed with angiogenic properties and were found to favor cell survival, invasion and growth [49]–[53]. Interestingly, endometriosis-like lesions developed in MIF-KO mice showed a significant down-regulation of BCL2, which promotes cell survival, but no noticeable change in the expression of the apoptotic BCL2-family member BAX, which binds to BCL2 and counteracts its apoptosis-preventing effects [54]. This makes plausible that an imbalance in the BCL2/BAX ratio may account for the reduced endometrial tissue growth observed in the absence of MIF. Moreover, PCNA immunostaining appeared to be decreased in KO/KO mice and WT/WT mice treated with ISO-1. These data further support MIF pro-survival effects, its possible involvement in endometriotic cell resistance to apoptosis and its relevance to endometriosis as numerous alterations in the apoptotic pathways were detected in women with endometriosis, including a significant BCL2/BAX disequilibrium favoring the BCL2 survival pathway [54]–[56].

Our current study also revealed a significant down-regulation of integrins αv and β3 in endometrial implants from MIF-KO mice. The integrin family of cell adhesion receptors regulates a diverse array of cellular functions including extracellular matrix (ECM) remodeling and cell migration, invasion and proliferation [57]. Integrins αv and β3 are also markers of active angiogenesis [58], [59], and showed an increased expression in endometriotic stromal cells [60]. This is consistent with our previous *in vitro* data showing the ability of MIF to upregulate αv and β3 integrins in endometrial cells and the increased expression of these factors in human endometriotic lesions, and provides the *in vivo* evidence that supports the involvement of MIF in the regulation and the abnormal expression of these main adhesion factors in endometriosis.

Cross experiments using endometrial tissue from MIF-KO mice injected into the peritoneal cavity of WT mice and *vice versa* further showed that the number and size of endometriosis-like lesions were significantly lower than those found in WT/WT mice, but significantly higher than that found in KO/KO mice. These findings suggest that MIF presence in both endometrial and peritoneal host tissues is required for ectopic endometrial tissue growth and point to the importance of endometrial-peritoneal interactions in the pathogenesis of endometriosis. The process by which endometrial cells attach to and invade host tissues remains unclear. Significant inflammatory changes in peritoneal tissue were found in women with endometriosis [61]–[63]. Several *in vivo* and *in vitro* models have been developed to study the role of endometrial-peritoneal interactions. The baboon has been proposed as an *in vivo* model to study endometrial-peritoneal implantation [64]. *In vitro*, potential interactions have been also studied [65], [66], but conclusions are controversial. To the best of our knowledge, this is the first report showing that conditions for such an interaction involve MIF. Ectopic implantation of endometrial tissue needs complex interactions between the host tissue and endometrial tissues. Further cellular and molecular studies are underway to define MIF exact role within endometrial-peritoneal interactions.

Several previous studies showed the advantage of targeting MIF for treating inflammatory conditions such as sepsis and asthma [67]. Therefore, our data using a MIF-KO and WT control mice models and showing for the first time that MIF may be required for ectopic endometrial tissue growth and progression of endometriosis lesions *in vivo*, argues in favor of the possible therapeutic interest for this molecule. Actually, there is a crucial need for non-steroid targeted treatment of endometriosis. The main current medical treatment of endometriosis is based on the suppression of ovarian function and estrogen production, which, however causes side effects and is associated with a high recurrence rate after treatment ends.
